# Towards Unraveling the Human Tooth Transcriptome: The Dentome

**DOI:** 10.1371/journal.pone.0124801

**Published:** 2015-04-07

**Authors:** Shijia Hu, Joel Parker, John Timothy Wright

**Affiliations:** 1 Pediatric Dentistry, University of North Carolina, Chapel Hill, North Carolina, United States of America; 2 Oral Biology Curriculum, University of North Carolina, Chapel Hill, North Carolina, United States of America; 3 Cancer Genetics, University of North Carolina, Chapel Hill, North Carolina, United States of America; Tufts University, UNITED STATES

## Abstract

The goal of the study was to characterize the transcriptome profiles of human ameloblasts and odontoblasts, evaluate molecular pathways and advance our knowledge of the human “dentome”. Laser capture microdissection was used to isolate odontoblasts and ameloblasts from human tooth buds (15-20week gestational age) from 4 fetuses. RNA was examined using Agilent 41k whole genome arrays at 2 different stages of enamel formation, presecretory and secretory. Probe detection was considered against the array negative control to control for background noise. Differential expression was examined using Significance Analysis of Microarrays (SAM) 4.0 between different cell types and developmental stages with a false discovery rate of 20%. Pathway analysis was conducted using Ingenuity Pathway Analysis software. We found that during primary tooth formation, odontoblasts expressed 14,802 genes, presecretory ameloblasts 15,179 genes and secretory ameloblasts 14,526 genes. Genes known to be active during tooth development for each cell type (eg *COL1A1*, *AMELX*) were shown to be expressed by our approach. Exploring further into the list of differentially expressed genes between the motile odontoblasts and non-motile presecretory ameloblasts we found several genes of interest that could be involved in cell movement (*FN1*, *LUM*, *ASTN1*). Furthermore, our analysis indicated that the Phospholipase C and ERK5 pathways, that are important for cell movement, were activated in the motile odontoblasts. In addition our pathway analysis identified *WNT3A* and *TGFB1* as important upstream contributors. Recent studies implicate these genes in the development of Schimke immuno-osseous dysplasia. The utility of laser capture microdissection can be a valuable tool in the examination of specific tissues or cell populations present in human tooth buds. Advancing our knowledge of the human dentome and related molecular pathways provides new insights into the complex mechanisms regulating odontogenesis and biomineralization. This knowledge could prove useful in future studies of odontogenic related pathologies.

## Introduction

Tooth formation or odontogenesis is strictly regulated at the molecular level and involves multiple complex processes including development of highly specialized cells that produce unique extracellular matrices and ultimately mineralized tissues including the hardest tissue in the body, enamel [1]. Ameloblasts, the cells that form enamel, undergo extensive histodifferentiation during their life cycle going from cuboidal to columnar to squamous morphologies while creating and regulating a unique and changing microenvironment and extracellular matrix [2,3]. During the process of producing a unique extracellular matrix, the ameloblasts move in a highly organized manner to produce enamel prisms that are directionally oriented into three dimensional patterns that are species specific [4].

Dentin forming odontoblasts, on the other hand, continue to lay down matrix and remain functional throughout the life of a tooth [5]. These cells are able to react to stimuli and lay down reparative or reactionary dentin when the tooth experiences environmental insults. During odontogenesis, ameloblasts, derived from the dental epithelium, are involved in molecular cross talk with the underlying mesenchymal cells that ultimately form odontoblasts [6]. Many of the molecular mechanisms involved in tooth formation and the specific genes and interactions that control odontogenesis remain unknown.

The roles of specific genes and pathways involved in tooth development have been queried by numerous investigators using the murine model [7–10]. Many human studies of odontogenesis have focused on single genes and pathways that are disease driven [11–13]. The study of human odontogenesis is challenging due to the issue of obtaining samples at different developmental stages and the difficulty in isolating the different tissue components of the developing tooth bud. Most research has been based on the examination of entire tooth buds [14,15] which does not allow interrogation of the disparate tissues present in a developing tooth.

Laser capture microdissection [16] allows the isolation of specific cells from microscopic regions of tissue samples [17,18]. Using this technique cells can be harvested from frozen sections or archival tissues embedded in paraffin [19,20]. As laser capture does not change or damage the target cell morphology and chemical content, it can be used for DNA, RNA or protein analyses. Recent development in microarray techniques and reduction in costs has led to novel approaches for the study of tissue and organ development [14,21]. Microarray technology allows examination of the the entire genome with very small samples thereby allowing targeted interrogation of gene expression. New bioinformatics approaches and the ability to examine entire pathways rather than individual genes is exciting as small changes in individual gene expression levels may be significant when examined in the context of overall pathway changes [22].

The objective of this study was to characterize the gene expression profiles of human ameloblasts and odontoblasts and to further unravel the transcriptome of human teeth that we call the dentome. The investigation reveals many genes and molecular pathways not previously known to be involved in tooth formation that appear to be important.

## Materials and Methods

### Tissue collection and preparation

Written consent was obtained from mothers that were preparing for elective abortions in this IRB approved investigation. Four human fetuses were obtained at ages 15–20 weeks gestation, immediately placed on ice and the tooth buds dissected from the jaws, placed in RNAlater and refrigerated at 4C for 1–4 weeks to allow decalcification by EDTA. The tissue was then frozen and stored at -80°C. The tissue was sectioned at -35C at a thickness of 7 microns and lightly stained with haematoxylin and eosin to allow better visualization of the different cell types. Only anterior teeth from both the maxillary and mandibular jaws were used due to similar stage of dental development.

### Laser capture of specific tissue

AutoPix automated LCM system from Arcturus (Arcturus Engineering, Santa Clara, CA, USA) was used to isolate the human odontoblasts and ameloblasts in different stages of enamel formation, using static image settings. Ameloblasts have different morphological features and organelle content during different stages of enamel formation. In this study ameloblasts were assigned to specific developmental stages based on the presence or absence of visible enamel matrix at the light microscope level and cell morphology (e.g. presence of Tomes Process). Cells isolated before enamel apposition were designated as being in the pre-secretory stage and if enamel extracellular matrix was visible the ameloblasts were classified as being in the secretory stage. Odontoblasts adjacent to the enamel epithelium secrete the predentin matrix and were harvested predominantly after some dentin matrix secretion. This process allowed standardization of the cell’s developmental stage despite slight differences in an individual tooth bud’s stage of development.

During the microdissection procedure, a CapSure cap is positioned over the tissue section. At the end of the LCM procedure the transparent thermoplastic film that covers the cap was peeled off with the attached cells of interest and placed into RNA extraction buffer. Images were obtained of the tissue sections before and after LCM, including the captured regions.

Total RNA was isolated from the microdissected cells with the PicoPure RNA Isolation kit (Arcturus Bioscience, Santa Clara, CA, USA). The quality and yield of total RNA were assessed on an Agilent Bioanalyzer 2100 (Agilent Technologies, Palo Alto, CA, USA). Samples from each cell type were then sent for analysis to determine the RNA Integrity Number (RIN).

### RNA Microarray

Four samples each of odontoblasts, pre-secretory ameloblasts and secretory ameloblasts from 4 fetuses were obtained and RNA was extracted. The RNA from each CapSure cap was separately isolated and RNAs from each individual tooth bud then pooled to obtain sufficient amounts of RNA. This gave us a total of 4 different samples of each cell type for microarray analysis. Whole genome human oligonucleotide microarrays (41K Agilent) were used to examine gene expression of the different tissue. The arrays contain 44K 60-mer oligonucleotides representing over 41K human genes and transcripts.

Two hundred nanograms of total RNA was converted into labeled cRNA with nucleotides coupled to fluorescent dye Cy3 using the Low RNA Input Linear Amplification Kit (Agilent Technologies, Palo Alto, CA) following the manufacturer’s protocol. The Human Universal Reference RNA from Stratagene (Santa Clara, CA, USA) was coupled with Cy5.

Cy3-labeled cRNA (1.65 ng) from each sample was hybridized to Agilent whole genome array 41k formated chips. The hybridized array was then washed, scanned and data was extracted from the scanned image using Feature Extraction version 9.5 (Agilent Technologies, Palo Alto, CA). The microarray data is then submitted to the Gene Expression Omnibus (GEO) microarray database (accession number GSE63289).

### RTPCR

RNA from a sample each of pre-secretory ameloblasts, secretory ameloblasts and odontoblasts were compared to the Human Universal Reference RNA from Stratagene by probing for high intensity, medium intensity and low intensity levels of expression based on the microarray data. The probes *ACTIN*, *AMELX*, *COL6A3*, *FAM40B*, *HPRT1*, *IL11* were selected based on intensity levels obtained from the microarrays. RTPCR was performed using QIAGEN RT2 qPCR Primer Assays (Frederick, MD, USA) in an Eppendorf Mastercycler gradient thermocycler. Experiments were repeated in triplicate. Intraclass correlation coefficient was calculated between microarray log ratio and RTPCR expression ratio using IBM SPSS 21.

### Data analysis

Unbiased cluster analysis was carried out on the samples using Cluster 3.0 and the heat maps visualized using Java TreeView-1.1.6r2.

For the expression data, each microarray was examined and the minimum intensity for expression was set at 95% confidence of the negative controls on that array. The lists were crossed referenced and only genes that were expressed in all 4 samples for each tissue type was determined to be expressed. Differential expression was examined using Significance analysis of microarrays (SAM) 4.0 between the different stages of ameloblasts and different tissues. The false discovery rates of the SAM analyses were set at 20%.

Ingenuity pathway analysis was used to examine the pathways of the differential expression between the samples specifically focusing on the upstream analysis.

## Results

We performed laser capture microdissection and microarray of 3 different cell types from tooth buds removed from 4 individual fetuses. Cells from each fetus were analyzed separately. According to the Agilent Bioanalyzer 2100 (Agilent Technologies, Palo Alto, CA, USA), the microdissection yielded an estimated 10–15 ng total RNA per sample with a 260/280 ratio between 2.12 and 2.14. The samples had an average RIN number of 5.4 ± 0.74 SD.

### Genes expressed during tooth formation

**Fig 1:**
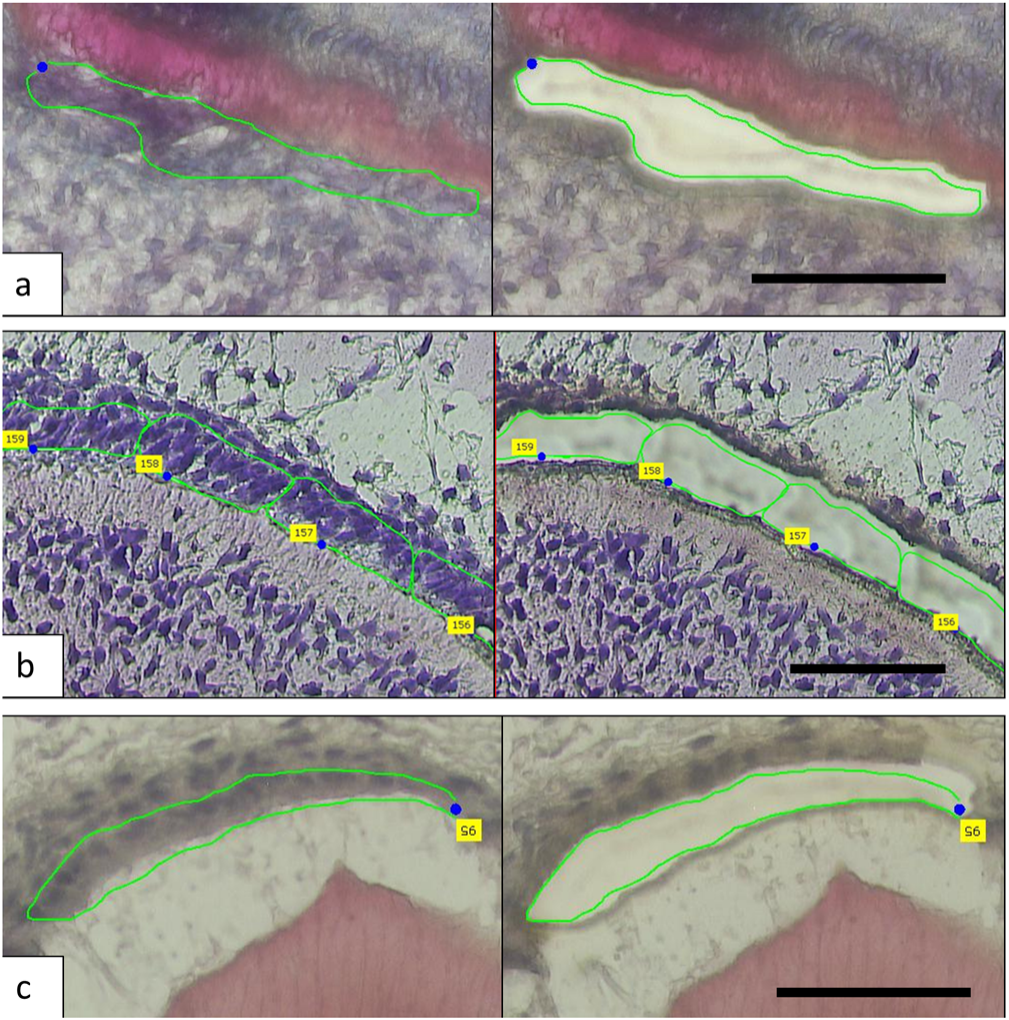
The micrographs show the laser capture of a—odontoblasts, b—pre-secretory ameloblasts and c—secretory ameloblasts. The left panel shows pre-capture and area to be captured while the right panel shows post-capture and the removal of target cells. Scale bar: 50 μm.

Laser capture microdissection allowed us to obtain small discrete areas of cells at relatively specific developmental stages from human tooth bud tissues (Fig 1). Using the negative control spots to eliminate background at 95% confidence level, we found that during tooth formation odontoblasts expressed 14,802 genes, pre-secretory ameloblast expresses 15,179 genes and secretory ameloblast expresses 14,526 genes.

### Differential gene expression in enamel and dentin formation

**Fig 2:**
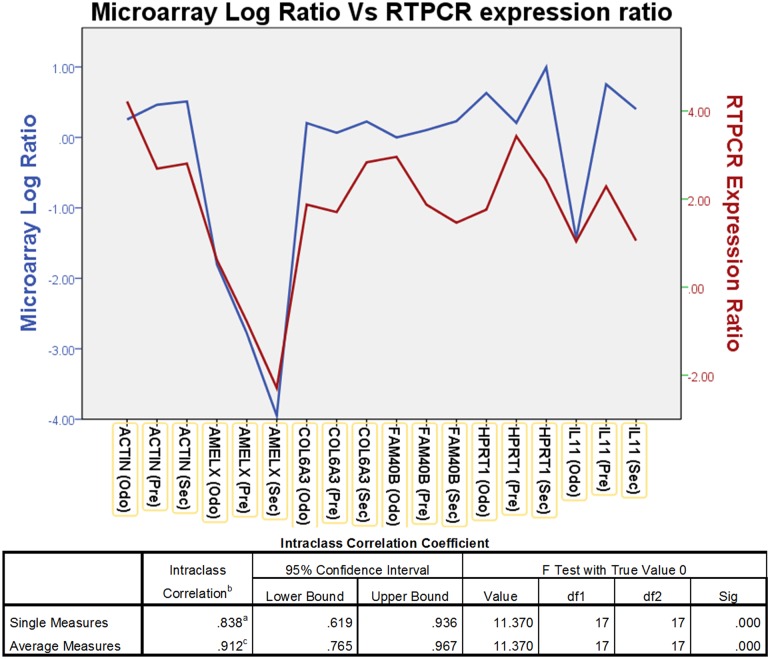
The graph shows comparison between the microarray Log ratio and RTPCR Log expression ratio. The microarray Log ratio is calculated using the ΔCt Method. Intraclass correlation coefficient analysis showed a significant correlation of 0.838 for single measure.

To validate the overall gene expression levels observed in the microarrays we performed RTPCR on selected genes in the samples and compared them with the standard reference RNA used in the microarray. The RTPCR analysis showed a intraclass correlation coefficient of 0.838 (p<0.05) for single measures, suggesting good correlation for genes (*ACTIN*, *AMELX*, *COL6A3*, *FAM40B*, *HPRT1*, *IL11*) that had high, moderate and low levels of expression on the microarray (Fig 2).

**Fig 3:**
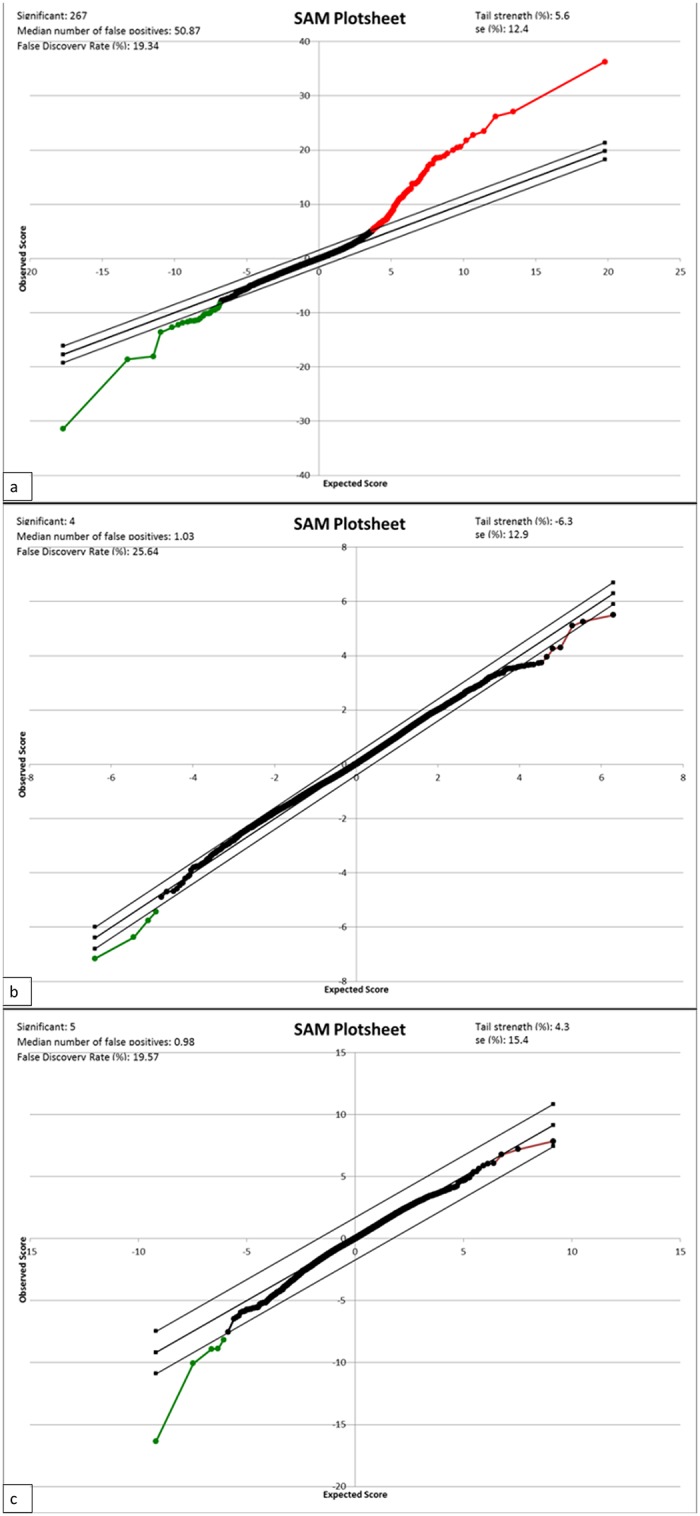
SAM plots: a—odontoblasts vs pre-secretory ameloblasts, b—odontoblast vs secretory ameloblasts, c—pre-secretory vs secretory ameloblasts. The red portion shows genes that are expressed at a higher level while the green portion shows genes that are expressed at a lower level with a FDR of 20%.

Using SAM analysis, we looked at differential gene expression between the different tissues at a false discovery rate of 20%. From the SAM plots (Fig 3) and unbiased cluster analysis (S1 Fig), we observed that the greatest gene expression difference was between odontoblasts and pre-secretory ameloblasts.

Odontoblasts had 131 genes expressed at significantly higher levels and 15 genes at lower levels compared with pre-secretory ameloblasts. In addition, 4 genes (*ENAM*, *ASTN1*, *AMELX*, *SEZ6L*) were expressed at lower levels in odontoblasts compared with secretory ameloblasts; 4 genes (*DMP1*, *AMBN*, *COPZ2*, *B3GALTL*) were expressed at lower levels in pre-secretory compared with secretory ameloblasts (S1 Table).

### Pathways important to enamel and dentin formation

The Ingenuity Pathway Analysis program uses an extensive database of canonical pathways to analyze the differentially expressed genes, the pathways they are involved in and examine the activated and/or inhibited pathways. Pathway analysis shows that the 2 networks with the greatest difference between odontoblasts and pre-secretory ameloblasts are mainly collagen and NF-KB driven. There were minimal differences for odontoblasts compared to secretory ameloblasts and pre-secretory compared to secretory ameloblasts (S2 Table).

Some of the canonical pathways that are different between odontoblasts and pre-secretory ameloblasts include—Intrinsic Prothrombin Activation Pathway, Hepatic Fibrosis / Hepatic Stellate Cell Activation, Atherosclerosis Signaling, Phospholipase C signaling, ERK5 signaling and Sphingosine-1-phosphate signaling (S2 Table).

### Upstream Analysis

**Fig 4:**
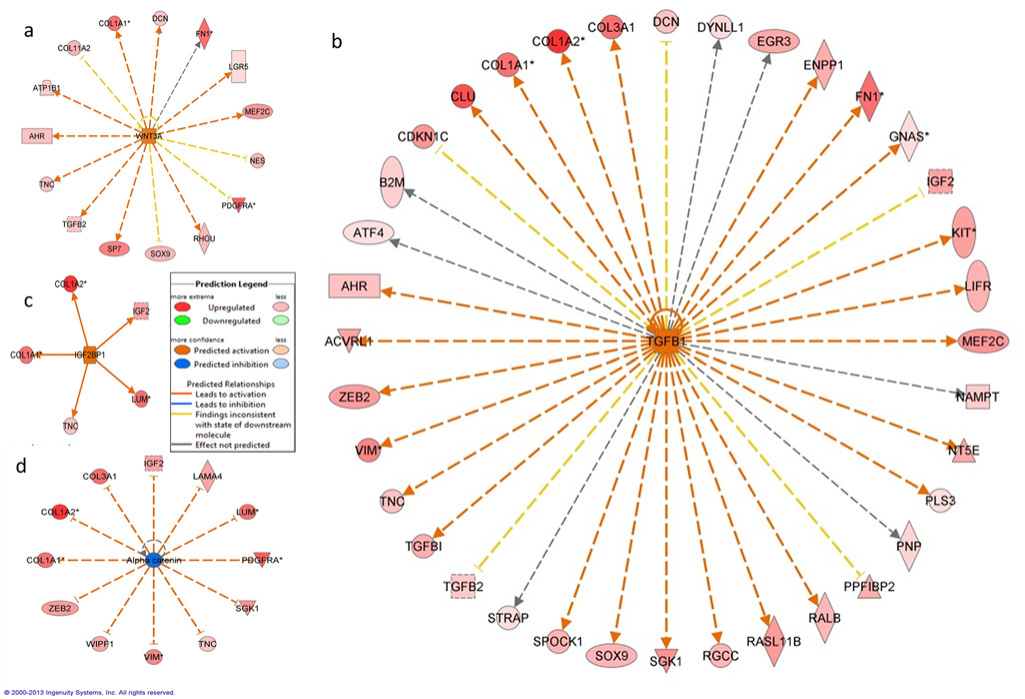
Upstream analysis: a—molecule network map for WNT3A, b—molecule network map for TGFB1, c—molecule network map for IGF2BP1, d—molecule network map for Alpha catenin. The predicted molecule is in the center with linkage to downstream targets based on differential expression from SAM analysis.

An upstream analysis examines current levels of gene expression detected by the microarray analysis and predicts which upstream regulators were most likely to be involved. Our analysis indicated that numerous upstream regulators were predicted to be different between odontoblasts and pre-secretory ameloblasts (S3 Table). Of note we found that *WNT3A* (Fig 4a), *TGFB1* (Fig 4b), *IGF2BP1* (Fig 4c), *SHH*, *GLI1* and *FGF2* were predicted to be significantly more active in odontoblast while Alpha catenin (Fig 4d) was inhibited in odontoblasts when compared with pre-secretory ameloblasts.

## Discussion

Examination of murine tooth development using many different approaches suggested that more than 300 genes are involved in the tooth formation [23]. More recently, the use of microarrays showed that 4362 genes are differentially expressed [24], suggesting that in fact large numbers of genes are involved. We showed that a much larger portion of the human genome is involved in the development of the human tooth. Many of the 14,802 genes and 15,179 genes expressed by odontoblasts and ameloblasts respectively are involved in basic cellular functions rather than tooth development specific functions. The present study clearly shows that the molecular control of odontogenesis is extremely complex and involves many more genes and molecular pathways than previously known.

Our observation that secretory ameloblast expression profile was more similar to odontoblast expression as compared with pre-secretory ameloblast was unexpected. This finding is likely due to secretory ameloblasts and odontoblasts sharing many similar characteristics as both are differentiated cells, are secreting an extracellular matrix, are controlling the microenvironment of that matrix, and are motile and moving away from the secreted matrix. This is in contrast to the pre-secretory ameloblasts that are in the process of differentiation, are not motile, and not secreting an extracellular matrix. As ameloblasts differentiate and mature, their expression profile becomes more similar to that of odontoblasts with both cells functioning to produce a mineralized extracellular matrix that involves similar yet distinct processes (Figs 3 and S1).

As expected, the SAM analysis showed significantly higher expression of known dentin products such as collagen type 1, collagen type 3 in odontoblasts when compared to pre-secretory ameloblasts. Our findings showed that the *ENAM* and *AMELX* genes which code for the enamel specific extracellular matrix proteins enamelin and amelogenin respectively were expressed at higher levels in enamel forming secretory ameloblasts (S1 Table). These differences in gene expression are reflective of the unique extracellular matrices produced by these different cell types and ultimately the compositionally and structurally different mineralized tissue they create.

Interestingly, fibronectin (*FN1*) and lumican (*LUM*), showed greater levels of expression in odontoblasts compared with pre-secretory ameloblasts. The proteins derived from these genes are thought to be involved in the organization of extracellular matrix [25,26] that is secreted by the odontoblasts during dentinogenesis. In addition, lumican is also shown to affect actin organization in the cell cytoskeleton [27] which has implications in the control of cell movement and shape. It is noteworthy that once the pre-secretory ameloblasts (non-motile cells) differentiate further to secretory ameloblasts (motile cells) *FN1* and *LUM* are not differently expressed compared with the odontoblasts (motile cells). Furthermore, *ASTN1*, which codes for the neuronal protein astrotactin, is expressed at a higher level in secretory ameloblasts compared to odontoblasts. Astrotactin is a major player in the migration and movement of neurons [28] and could be an important component in the control of ameloblast movement, potentially reflecting the ectodermal origin of ameloblasts and conservation of this protein function during enamel formation. There is virtually nothing known about the complex regulation of motility in these different odontogenic cell types but it is known that motility is critical in the normal formation and structure of the mineralized dentin and enamel. The other differentially expressed genes have been implicated in various other pathologies such as Peters' plus syndrome with cleft lip/palate (B3GALTL) [29], tumor formation (COPZ2) [30] and lung cancer development (SEZ6L) [31] but have not yet been associated with tooth development.

Not surprisingly, the top canonical pathways are driven by clusters of collagen genes (Intrinsic Prothrombin Activation Pathway, Hepatic Fibrosis / Hepatic Stellate Cell Activation, Atherosclerosis Signaling) (S2 Table). Of note, the Phospholipase C signaling pathway is an important signal transduction pathway that is implicated in cancer development [32] and regulates chemotaxis through the modulation of Phosphoinositide 3-kinase activity to provide a direction-sensing machinery [33]. In addition, the activation of ERK5 signaling pathway is associated with disruption to cell actin cytoskeleton. This change in cell actin dynamics can lead to increased cell motility and decreased cell adhesion [34]. Furthermore, Sphingosine-1-phosphate is thought to be a more potent chemoattractant of dental pulp stem cells than transforming growth factor β1 (TGF-β1), fibroblast growth factors (FGFs), and epidermal growth factors (EGFs) [35]. Dental pulp stem cells are postulated to form reparative dentin in the event of tooth injury by migrating and differentiating into odontoblasts [36]. The signaling pathways identified in this study could be contributing to the motility of odontoblasts compared to non-motile pre-secretory ameloblasts.

Examination of upstream regulators showed that *SHH*, *GLI1*, *FGF2* and *TGFB1* are likely involved in dentinogenesis. This finding was expected as these genes have been well characterized to be involved in tooth formation and the disturbances of these genes are known to lead to tooth malformation and agenesis [37–39]. Upstream analysis also indicated that both *WNT3A* and *TGFB1* are activated in odontoblasts (Fig 4a and Fig 4b). Recent investigation of Schimke immuno-osseous dysplasia, a syndrome associated with mutations of the *SMARCAL1* gene shows there are cellular disturbance in the expression of *WNT3A* and *TGFB1* as a result of abnormal *SMARCAL1*. Patients with this rare condition exhibit microdontia, hypodontia and severe molar root hypoplasia [40]. These findings suggest that these pathways could be important in root formation and tooth morphogenesis.

The present study also found that *IGF2BP1* was identified as an active upstream regulator in odontoblasts (Fig 4c) and a human GWAS showed that it is one of the loci associated with tooth agenesis [41]. *IGF2BP1* has also been implicated in the upregulation of betaTrCP1 leading to the activation of beta-catenin/Tcf signaling [42] that is important in beta-actin mRNA translation and cell migration. This pathway provides a possible mechanism for the movement of odontoblasts as it deposits newly formed dentin.

Conversely, Alpha catenin, that was predicted to be expressed at a lower level in odontoblasts when compared to pre-secretory ameloblasts (Fig 4d), is known to be important in ameloblast development [43]. The cadherin–catenin complex, with Wnt/beta-catenin signaling, also has critical roles in regulating cell motility/adhesion [44]. The nature and complexity of movement has been shown to be quite different between odontoblasts and ameloblasts. A recent paper described the importance of MMP20 and the cadherin complexes during ameloblast maturation to allow movement of the epithelial cells by the switching of cadherin types [45].

One of the shortcomings of this study is that we were only able to obtain primary tooth buds for examination. It is likely that there are differences between the dentome of primary and permanent teeth; however, permanent tooth buds were not available in our study sample. Future studies of permanent tooth buds using similar protocols will shed light on the differences between the dentome of primary and permanent teeth. Later stages of tooth formation were not evaluated as the later stages of tooth formation (e.g. maturation) were not available. In addition, although the use of the LCM aids in providing a relatively discrete sample, there is still a possibility of contamination from adjacent cell layers such as the stratum intermedium collected with the ameloblasts. Furthermore, we did not analyze cementum or root forming cells, leaving the dentome incomplete at the moment.

In summary, our results support the utility of laser capture microdissection as a valuable tool that allows interrogation of different tissues and cell types present in human teeth during different stages of development. The use of laser capture and RNA microarrays shows that the early developing human tooth transcriptome involves more genes than anticipated and diverse molecular pathways that are differentially activated in the tooth forming cells. We identified genes and pathways not previously known to play a role in tooth formation. For example, the genes and pathways involved in cell movement may be essential processes in normal odontogenesis that are worth closer examination. Unravelling the human dentome will advance our knowledge of tooth formation and the critical events that are regulated by gene expression and that control cell function and development of the tooth.

## Supporting Information

S1 Fig Heat map from cluster analysis. The map shows clustering of genes associated with collagen and extra cellular matrix formation.
(TIF)

S1 Table Significantly different genes from SAM analysis. Note that some genes are repeated as there were multiple probes for those genes in the microarray
(DOCX)

S2 Table Top 20 differentially expressed canonical pathways between odontoblasts and pre-secretory ameloblasts. (DOCX)

S3 Table Top 20 different upstream regulators between odontoblasts and pre-secretory ameloblasts. (DOCX)

## References

[1] Thesleff I, Keranen S, Jernvall J (2001) Enamel knots as signaling centers linking tooth morphogenesis and odontoblast differentiation. Adv Dent Res 15: 14–18. 12640732 10.1177/08959374010150010401

[2] Deutsch D, Catalano-Sherman J, Dafni L, David S, Palmon A (1995) Enamel matrix proteins and ameloblast biology. Connect Tissue Res 32: 97–107. 7554940 10.3109/03008209509013710

[3] Simmer JP, Richardson AS, Hu YY, Smith CE, Ching-Chun Hu J (2012) A post-classical theory of enamel biomineralization… and why we need one. Int J Oral Sci 4: 129–134. 10.1038/ijos.2012.59 22996272 PMC3464985

[4] Bartlett JD, Smith CE (2013) Modulation of cell-cell junctional complexes by matrix metalloproteinases. J Dent Res 92: 10–17. 10.1177/0022034512463397 23053846 PMC3521448

[5] Couve E, Osorio R, Schmachtenberg O (2013) The amazing odontoblast: activity, autophagy, and aging. J Dent Res 92: 765–772. 10.1177/0022034513495874 23803461

[6] Jernvall J, Thesleff I (2000) Reiterative signaling and patterning during mammalian tooth morphogenesis. Mech Dev 92: 19–29. 10704885 10.1016/s0925-4773(99)00322-6

[7] Kim KM, Lim J, Choi YA, Kim JY, Shin HI, Park EK (2012) Gene expression profiling of oral epithelium during tooth development. Arch Oral Biol 57: 1100–1107. 10.1016/j.archoralbio.2012.02.019 22417879

[8] D'Souza RN, Aberg T, Gaikwad J, Cavender A, Owen M, Karsenty G, et al (1999) Cbfa1 is required for epithelial-mesenchymal interactions regulating tooth development in mice. Development 126: 2911–2920. 10357935 10.1242/dev.126.13.2911

[9] Jarvinen E, Salazar-Ciudad I, Birchmeier W, Taketo MM, Jernvall J, Thesleff I (2006) Continuous tooth generation in mouse is induced by activated epithelial Wnt/beta-catenin signaling. Proc Natl Acad Sci U S A 103: 18627–18632. 17121988 10.1073/pnas.0607289103 PMC1693713

[10] Dassule HR, McMahon AP (1998) Analysis of epithelial-mesenchymal interactions in the initial morphogenesis of the mammalian tooth. Dev Biol 202: 215–227. 9769173 10.1006/dbio.1998.8992

[11] Bergendal B, Klar J, Stecksen-Blicks C, Norderyd J, Dahl N (2011) Isolated oligodontia associated with mutations in EDARADD, AXIN2, MSX1, and PAX9 genes. Am J Med Genet A 155A: 1616–1622. 10.1002/ajmg.a.34045 21626677

[12] Liu F, Millar SE (2010) Wnt/beta-catenin signaling in oral tissue development and disease. J Dent Res 89: 318–330. 10.1177/0022034510363373 20200414 PMC3140915

[13] Rufini A, Barlattani A, Docimo R, Velletri T, Niklison-Chirou MV, Agostini M, et al (2011) p63 in tooth development. Biochem Pharmacol 82: 1256–1261. 10.1016/j.bcp.2011.07.068 21787761

[14] Heikinheimo K, Jee KJ, Niini T, Aalto Y, Happonen RP, Leivo I, et al (2002) Gene expression profiling of ameloblastoma and human tooth germ by means of a cDNA microarray. J Dent Res 81: 525–530. 12147741 10.1177/154405910208100805

[15] Lin D, Huang Y, He F, Gu S, Zhang G, Chen Y, et al (2007) Expression survey of genes critical for tooth development in the human embryonic tooth germ. Dev Dyn 236: 1307–1312. 17394220 10.1002/dvdy.21127

[16] Decarlo K, Emley A, Dadzie OE, Mahalingam M (2011) Laser capture microdissection: methods and applications. Methods Mol Biol 755: 1–15. 10.1007/978-1-61779-163-5_1 21761290

[17] Sun JX, Horst OV, Bumgarner R, Lakely B, Somerman MJ, Zhang H (2012) Laser capture microdissection enables cellular and molecular studies of tooth root development. Int J Oral Sci 4: 7–13. 10.1038/ijos.2012.15 22422086 PMC3412663

[18] Chokechanachaisakul U, Kaneko T, Yamanaka Y, Okiji T, Suda H (2012) A novel whole tooth-in-jaw-bone culture of rat molars: morphological, immunohistochemical, and laser capture microdissection analysis. Microsc Res Tech 75: 1341–1347. 10.1002/jemt.22072 22623030

[19] Hayashi Y, Matsunaga T, Yamamoto G, Nishii K, Usui M, Yamamoto M, et al (2010) Comprehensive analysis of gene expression in the junctional epithelium by laser microdissection and microarray analysis. J Periodontal Res 45: 618–625. 10.1111/j.1600-0765.2010.01276.x 20546111

[20] Salmon CR, Silverio KG, Giorgetti AP, Sallum EA, Casati MZ, Nociti FH Jr. (2012) Gene expression analysis in microdissected samples from decalcified tissues. Diagn Mol Pathol 21: 120–126. 10.1097/PDM.0b013e31823e9395 22555095

[21] Tranasi M, Sberna MT, Zizzari V, D'Apolito G, Mastrangelo F, Salini L, et al (2009) Microarray evaluation of age-related changes in human dental pulp. J Endod 35: 1211–1217. 10.1016/j.joen.2009.05.026 19720218

[22] Ganter B, Zidek N, Hewitt PR, Muller D, Vladimirova A (2008) Pathway analysis tools and toxicogenomics reference databases for risk assessment. Pharmacogenomics 9: 35–54. 18154447 10.2217/14622416.9.1.35

[23] Nieminen P, Pekkanen M, Aberg T, Thesleff I (1998) A graphical WWW-database on gene expression in tooth. Eur J Oral Sci 106 Suppl 1: 7–11. 9541196 10.1111/j.1600-0722.1998.tb02146.x

[24] Landin MA, Shabestari M, Babaie E, Reseland JE, Osmundsen H (2012) Gene Expression Profiling during Murine Tooth Development. Front Genet 3: 139 10.3389/fgene.2012.00139 22866057 PMC3408794

[25] Matheson S, Larjava H, Hakkinen L (2005) Distinctive localization and function for lumican, fibromodulin and decorin to regulate collagen fibril organization in periodontal tissues. J Periodontal Res 40: 312–324. 15966909 10.1111/j.1600-0765.2005.00800.x

[26] Kadler KE, Hill A, Canty-Laird EG (2008) Collagen fibrillogenesis: fibronectin, integrins, and minor collagens as organizers and nucleators. Curr Opin Cell Biol 20: 495–501. 10.1016/j.ceb.2008.06.008 18640274 PMC2577133

[27] Radwanska A, Baczynska D, Nowak D, Brezillon S, Popow A, Maquart FX, et al (2008) Lumican affects actin cytoskeletal organization in human melanoma A375 cells. Life Sci 83: 651–660. 10.1016/j.lfs.2008.09.008 18848571

[28] Wilson PM, Fryer RH, Fang Y, Hatten ME (2010) Astn2, a novel member of the astrotactin gene family, regulates the trafficking of ASTN1 during glial-guided neuronal migration. J Neurosci 30: 8529–8540. 10.1523/JNEUROSCI.0032-10.2010 20573900 PMC2905051

[29] Schoner K, Kohlhase J, Muller AM, Schramm T, Plassmann M, Schmitz R, et al (2013) Hydrocephalus, agenesis of the corpus callosum, and cleft lip/palate represent frequent associations in fetuses with Peters' plus syndrome and B3GALTL mutations. Fetal PPS phenotypes, expanded by Dandy Walker cyst and encephalocele. Prenat Diagn 33: 75–80. 10.1002/pd.4012 23161355

[30] Shtutman M, Baig M, Levina E, Hurteau G, Lim CU, Broude E, et al (2011) Tumor-specific silencing of COPZ2 gene encoding coatomer protein complex subunit zeta 2 renders tumor cells dependent on its paralogous gene COPZ1. Proc Natl Acad Sci U S A 108: 12449–12454. 10.1073/pnas.1103842108 21746916 PMC3145676

[31] Gorlov IP, Meyer P, Liloglou T, Myles J, Boettger MB, Cassidy A, et al (2007) Seizure 6-like (SEZ6L) gene and risk for lung cancer. Cancer Res 67: 8406–8411. 17804757 10.1158/0008-5472.CAN-06-4784

[32] Bunney TD, Katan M (2010) Phosphoinositide signalling in cancer: beyond PI3K and PTEN. Nat Rev Cancer 10: 342–352. 10.1038/nrc2842 20414202

[33] Kolsch V, Charest PG, Firtel RA (2008) The regulation of cell motility and chemotaxis by phospholipid signaling. J Cell Sci 121: 551–559. 10.1242/jcs.023333 18287584 PMC2671295

[34] Barros JC, Marshall CJ (2005) Activation of either ERK1/2 or ERK5 MAP kinase pathways can lead to disruption of the actin cytoskeleton. J Cell Sci 118: 1663–1671. 15797923 10.1242/jcs.02308

[35] Howard C, Murray PE, Namerow KN (2010) Dental pulp stem cell migration. J Endod 36: 1963–1966. 10.1016/j.joen.2010.08.046 21092813

[36] Qvist V (1975) Pulp reactions in human teeth to tooth colored filling materials. Scand J Dent Res 83: 54–66. 1056085 10.1111/j.1600-0722.1975.tb00420.x

[37] Cobourne MT, Hardcastle Z, Sharpe PT (2001) Sonic hedgehog regulates epithelial proliferation and cell survival in the developing tooth germ. J Dent Res 80: 1974–1979. 11759005 10.1177/00220345010800110501

[38] Pispa J, Jung HS, Jernvall J, Kettunen P, Mustonen T, Tabata MJ, et al (1999) Cusp patterning defect in Tabby mouse teeth and its partial rescue by FGF. Dev Biol 216: 521–534. 10642790 10.1006/dbio.1999.9514

[39] Lee DS, Yoon WJ, Cho ES, Kim HJ, Gronostajski RM, Cho MI, et al (2011) Crosstalk between nuclear factor I-C and transforming growth factor-beta1 signaling regulates odontoblast differentiation and homeostasis. PLoS One 6: e29160 10.1371/journal.pone.0029160 22195013 PMC3241690

[40] Morimoto M, Kerouredan O, Gendronneau M, Shuen C, Baradaran-Heravi A, Asakura Y, et al (2012) Dental abnormalities in Schimke immuno-osseous dysplasia. J Dent Res 91: 29S–37S. 22699664 10.1177/0022034512450299 PMC3383106

[41] Pillas D, Hoggart CJ, Evans DM, O'Reilly PF, Sipila K, Lahdesmaki R, et al (2010) Genome-wide association study reveals multiple loci associated with primary tooth development during infancy. PLoS Genet 6: e1000856 10.1371/journal.pgen.1000856 20195514 PMC2829062

[42] Noubissi FK, Elcheva I, Bhatia N, Shakoori A, Ougolkov A, Liu J, et al (2006) CRD-BP mediates stabilization of betaTrCP1 and c-myc mRNA in response to beta-catenin signalling. Nature 441: 898–901. 16778892 10.1038/nature04839

[43] Sorkin BC, Wang MY, Dobeck JM, Albergo KL, Skobe Z (2000) The cadherin-catenin complex is expressed alternately with the adenomatous polyposis coli protein during rat incisor amelogenesis. J Histochem Cytochem 48: 397–406. 10681393 10.1177/002215540004800309

[44] Van den Bossche J, Malissen B, Mantovani A, De Baetselier P, Van Ginderachter JA (2012) Regulation and function of the E-cadherin/catenin complex in cells of the monocyte-macrophage lineage and DCs. Blood 119: 1623–1633. 10.1182/blood-2011-10-384289 22174153

[45] Guan X, Bartlett JD (2013) MMP20 modulates cadherin expression in ameloblasts as enamel develops. J Dent Res 92: 1123–1128. 10.1177/0022034513506581 24067343 PMC3834655

