# Supplementary figures and images for: Towards Unraveling the Human Tooth Transcriptome: The Dentome
Source: PLoS One. 2015 Apr 7;10(4):e0124801. doi: 10.1371/journal.pone.0124801 (PMC4388651; doi:10.1371/journal.pone.0124801)

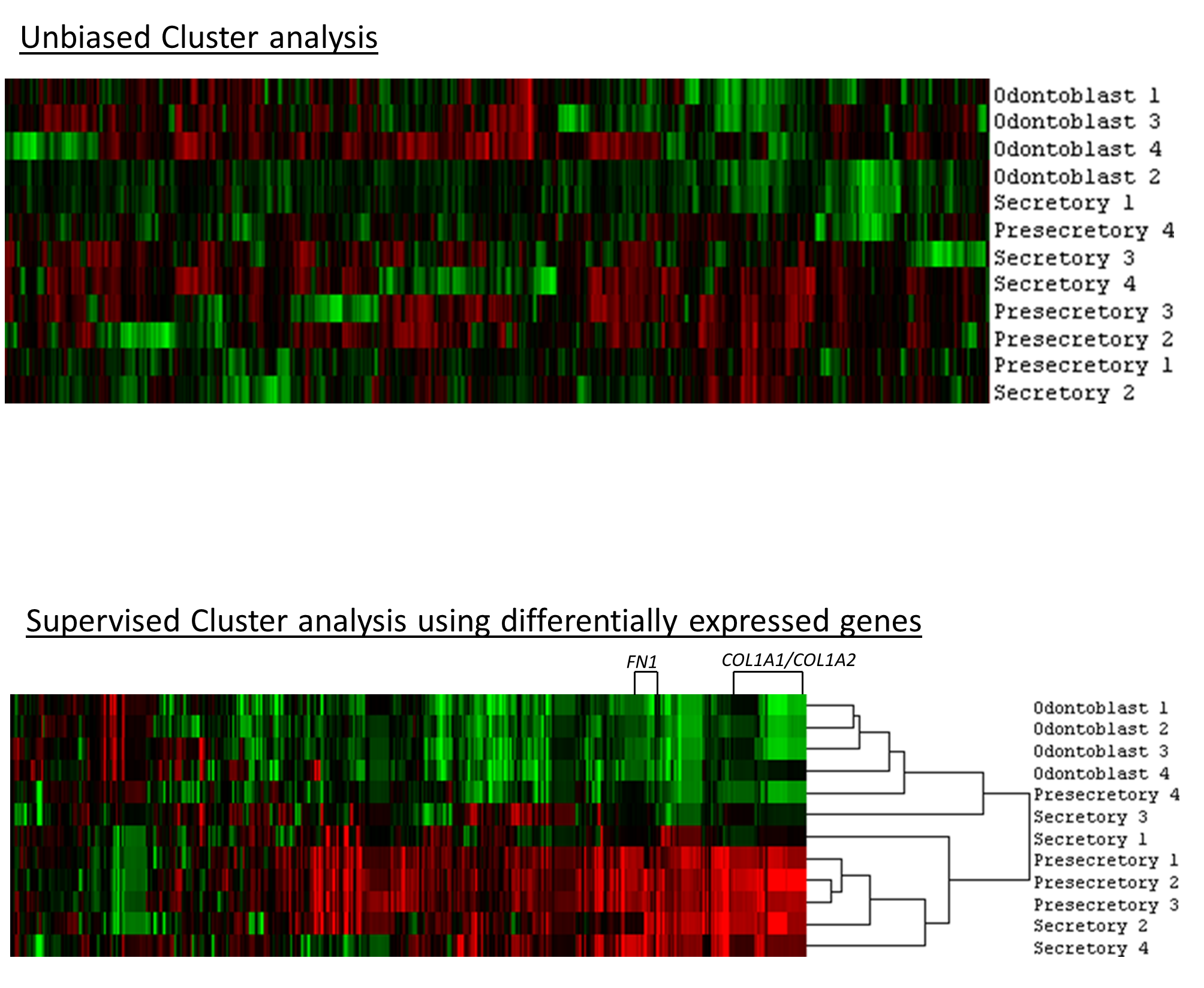

Supplement: S1 Fig — The map shows clustering of genes associated with collagen and extra cellular matrix formation. (TIF) [file pone.0124801.s001.tif]
