# Supplementary material for: Towards Unraveling the Human Tooth Transcriptome: The Dentome
Source: PLoS One. 2015 Apr 7;10(4):e0124801. doi: 10.1371/journal.pone.0124801 (PMC4388651; doi:10.1371/journal.pone.0124801)
Supplement: S1 Table — Note that some genes are repeated as there were multiple probes for those genes in the microarray (DOCX) [file pone.0124801.s002.docx]

**S1 Table. Significantly different genes from SAM analysis.** Note that some genes are repeated as there were multiple probes for those genes in the microarray

| **Odontoblasts vs pre-secretory ameloblasts** | | | |
| --- | --- | --- | --- |
| **Positive genes (131 genes and 241 probes)** | | | |
| **Gene ID** | **Gene Name** | **Fold Change** | **q-value(%)** |
| NM_000089 | COL1A2 | 8.548655178 | 18.90170085 |
| NM_000089 | COL1A2 | 8.253386022 | 18.01968985 |
| NM_000089 | COL1A2 | 7.943128049 | 18.01968985 |
| NM_000089 | COL1A2 | 7.81257606 | 18.01968985 |
| NM_000089 | COL1A2 | 7.778758475 | 18.01968985 |
| NM_000089 | COL1A2 | 7.684403934 | 17.40088182 |
| NM_000089 | COL1A2 | 7.613167242 | 17.40088182 |
| NM_000089 | COL1A2 | 7.54436376 | 17.40088182 |
| NM_203339 | CLU | 7.331915649 | 0 |
| NM_000089 | COL1A2 | 7.183942366 | 19.34106852 |
| NM_000089 | COL1A2 | 7.0040606 | 16.90104045 |
| AK021543 | LOC100128178 | 6.7489885 | 16.39461008 |
| XM_001718912 | LOC100128178 | 6.725479392 | 2.274911011 |
| AA599881 | PDGFRA | 6.671561986 | 4.733282588 |
| BX427588 | BX427588 | 6.637844268 | 14.36263262 |
| NM_017633 | FAM46A | 6.628734995 | 2.274911011 |
| BE710245 | BE710245 | 6.229470087 | 0 |
| NM_000090 | COL3A1 | 6.069819149 | 14.36263262 |
| CA418661 | CA418661 | 6.054524524 | 2.274911011 |
| NM_212482 | FN1 | 5.999441577 | 0 |
| Z74615 | COL1A1 | 5.951508471 | 18.01968985 |
| NM_032229 | SLITRK6 | 5.92571644 | 16.39461008 |
| NM_212482 | FN1 | 5.901809667 | 0 |
| Z74615 | COL1A1 | 5.901180477 | 18.90170085 |
| NM_212482 | FN1 | 5.891382475 | 0 |
| Z74615 | COL1A1 | 5.879023263 | 18.90170085 |
| Z74615 | COL1A1 | 5.845019415 | 18.01968985 |
| Z74615 | COL1A1 | 5.838422373 | 18.01968985 |
| Z74615 | COL1A1 | 5.799260717 | 18.90170085 |
| Z74615 | COL1A1 | 5.796797137 | 18.01968985 |
| NM_212482 | FN1 | 5.742431921 | 0 |
| Z74615 | COL1A1 | 5.72132047 | 18.01968985 |
| Z74615 | COL1A1 | 5.708118458 | 18.01968985 |
| Z74615 | COL1A1 | 5.648826326 | 18.90170085 |
| NM_212482 | FN1 | 5.646277441 | 0 |
| NM_002345 | LUM | 5.483308934 | 12.11119291 |
| NM_212482 | FN1 | 5.348849376 | 0 |
| NM_017821 | RHBDL2 | 5.338564555 | 17.40088182 |
| AY665468 | RASSF8 | 5.271583578 | 2.274911011 |
| NM_212482 | FN1 | 5.215213191 | 3.432321877 |
| NM_212482 | FN1 | 5.178529894 | 0 |
| NM_152588 | TMTC2 | 5.169203528 | 2.274911011 |
| NM_212482 | FN1 | 5.162499723 | 3.432321877 |
| NM_152860 | SP7 | 5.159489351 | 4.95297081 |
| NM_007211 | RASSF8 | 5.106462161 | 4.95297081 |
| NM_003380 | VIM | 5.09932909 | 16.39461008 |
| ENST00000369519 | RBM20 | 4.933614822 | 16.90104045 |
| NM_138440 | VASN | 4.906250758 | 3.432321877 |
| NM_212482 | FN1 | 4.883590579 | 0 |
| AW276332 | AW276332 | 4.812147976 | 5.754186676 |
| A_24_P585198 | A_24_P585198 | 4.694928267 | 4.733282588 |
| NM_018667 | SMPD3 | 4.639068612 | 8.325206255 |
| NM_006206 | PDGFRA | 4.607681028 | 0 |
| A_32_P122492 | A_32_P122492 | 4.590696533 | 16.39461008 |
| NM_016519 | AMBN | 4.578726591 | 2.274911011 |
| NM_005627 | SGK1 | 4.540742173 | 12.98511153 |
| NM_032777 | GPR124 | 4.459142226 | 3.432321877 |
| NM_002397 | MEF2C | 4.414035534 | 4.733282588 |
| NM_007211 | RASSF8 | 4.395002263 | 5.754186676 |
| NM_002448 | MSX1 | 4.387704852 | 16.39461008 |
| NM_000076 | CDKN1C | 4.380678718 | 16.39461008 |
| A_32_P1076 | A_32_P1076 | 4.369168852 | 4.95297081 |
| NM_001080393 | GLT8D4 | 4.368411174 | 4.95297081 |
| A_24_P934434 | A_24_P934434 | 4.304067698 | 17.40088182 |
| THC2537367 | THC2537367 | 4.288640443 | 14.87146219 |
| NM_032606 | CAPS2 | 4.246479256 | 12.22764669 |
| NM_001015880 | PAPSS2 | 4.239382329 | 3.432321877 |
| NM_023940 | RASL11B | 4.225946757 | 3.432321877 |
| NM_018409 | LRP2BP | 4.153927115 | 16.90104045 |
| NM_014795 | ZEB2 | 4.14280945 | 12.22764669 |
| NM_031302 | GLT8D2 | 4.091551536 | 12.11119291 |
| THC2698311 | THC2698311 | 4.082724761 | 17.40088182 |
| ENST00000301171 | ENST00000301171 | 4.027322326 | 4.95297081 |
| NM_000222 | KIT | 3.938961239 | 0 |
| NM_000222 | KIT | 3.92793762 | 0 |
| NM_000222 | KIT | 3.90437791 | 0 |
| NM_005014 | OMD | 3.866091843 | 14.36263262 |
| THC2515368 | THC2515368 | 3.842612706 | 0 |
| NM_153605 | DKFZp667G2110 | 3.842221383 | 19.34106852 |
| NM_001931 | DLAT | 3.84181076 | 16.39461008 |
| NM_000222 | KIT | 3.836628458 | 0 |
| NM_000222 | KIT | 3.835772467 | 0 |
| NM_000020 | ACVRL1 | 3.835072841 | 0 |
| CR613436 | CR613436 | 3.831032783 | 16.90104045 |
| NM_000612 | IGF2 | 3.816271288 | 18.90170085 |
| NM_004563 | PCK2 | 3.815180005 | 14.36263262 |
| NM_016613 | C4orf18 | 3.794037438 | 14.87146219 |
| THC2648759 | THC2648759 | 3.787783973 | 3.432321877 |
| NM_004065 | CDR1 | 3.760905162 | 8.325206255 |
| NM_000222 | KIT | 3.753540357 | 0 |
| NM_002290 | LAMA4 | 3.747692044 | 4.95297081 |
| NM_000222 | KIT | 3.742886973 | 0 |
| NM_054034 | FN1 | 3.698444983 | 0 |
| NM_001077269 | WIPF1 | 3.697885728 | 16.39461008 |
| NM_194318 | B3GALTL | 3.678118631 | 2.274911011 |
| NM_004430 | EGR3 | 3.633812464 | 12.98511153 |
| NM_018431 | DOK5 | 3.632108836 | 16.39461008 |
| NM_138455 | CTHRC1 | 3.623494147 | 4.95297081 |
| NM_002526 | NT5E | 3.606928816 | 7.524705653 |
| NM_152721 | DOK6 | 3.596877872 | 14.36263262 |
| CN312045 | CN312045 | 3.576454223 | 16.39461008 |
| CA840930 | CA840930 | 3.575519007 | 7.524705653 |
| NM_000222 | KIT | 3.554511749 | 0 |
| A_24_P144314 | A_24_P144314 | 3.548647471 | 17.40088182 |
| NM_001451 | FOXF1 | 3.544360304 | 4.95297081 |
| NM_015036 | ENDOD1 | 3.518230798 | 19.34106852 |
| NM_000222 | KIT | 3.486914249 | 0 |
| NM_003380 | VIM | 3.480559655 | 4.95297081 |
| NM_000358 | TGFBI | 3.434077068 | 12.11119291 |
| AK054921 | CDR1 | 3.407561199 | 5.754186676 |
| NM_019035 | PCDH18 | 3.388007193 | 4.95297081 |
| NM_006208 | ENPP1 | 3.387263809 | 2.274911011 |
| NM_003621 | PPFIBP2 | 3.364798956 | 16.39461008 |
| NM_000222 | KIT | 3.347526944 | 2.274911011 |
| NM_053044 | HTRA3 | 3.337063519 | 14.36263262 |
| AF116687 | AF116687 | 3.297046089 | 16.39461008 |
| NM_000559 | HBG1 | 3.283150385 | 4.95297081 |
| NM_014458 | KLHL20 | 3.215343292 | 4.95297081 |
| NM_022832 | USP46 | 3.196391456 | 19.34106852 |
| NM_002310 | LIFR | 3.185960383 | 16.39461008 |
| NM_003596 | TPST1 | 3.172933365 | 14.36263262 |
| NM_080680 | COL11A2 | 3.166020306 | 18.01968985 |
| CR621244 | PWP1 | 3.146922049 | 17.40088182 |
| NM_000559 | HBG1 | 3.125854478 | 3.432321877 |
| BF955364 | BF955364 | 3.121565386 | 7.524705653 |
| NM_012388 | PLDN | 3.105303154 | 16.39461008 |
| NM_000559 | HBG1 | 3.101443056 | 2.274911011 |
| NM_014059 | C13orf15 | 3.090701001 | 18.90170085 |
| NM_000559 | HBG1 | 3.075286646 | 3.432321877 |
| NM_000346 | SOX9 | 3.071574825 | 7.524705653 |
| NM_000559 | HBG1 | 3.055493051 | 4.95297081 |
| NM_006617 | NES | 3.029191763 | 14.36263262 |
| NM_000559 | HBG1 | 3.018087991 | 2.274911011 |
| NM_002881 | RALB | 3.009639271 | 14.87146219 |
| NM_052885 | SLC2A13 | 2.966543452 | 14.36263262 |
| NM_000559 | HBG1 | 2.955514249 | 3.432321877 |
| NM_021205 | RHOU | 2.937552138 | 4.95297081 |
| NM_000559 | HBG1 | 2.933090827 | 2.274911011 |
| NM_000559 | HBG1 | 2.928898648 | 2.274911011 |
| AL573456 | TGFBI | 2.907208447 | 18.90170085 |
| NM_000559 | HBG1 | 2.89560184 | 0 |
| NM_016205 | PDGFC | 2.893554 | 16.90104045 |
| NM_000559 | HBG1 | 2.88207739 | 2.274911011 |
| NM_002968 | SALL1 | 2.875415137 | 19.34106852 |
| NM_002961 | S100A4 | 2.80309243 | 14.36263262 |
| AI133411 | AI133411 | 2.733139594 | 2.274911011 |
| NM_002338 | LSAMP | 2.714900597 | 14.87146219 |
| NM_004294 | MTRF1 | 2.642485907 | 18.90170085 |
| NM_005065 | SEL1L | 2.63702887 | 14.87146219 |
| NM_013261 | PPARGC1A | 2.634348719 | 19.34106852 |
| NM_001920 | DCN | 2.577269268 | 14.36263262 |
| ENST00000381298 | ENST00000381298 | 2.575847997 | 18.01968985 |
| NM_001621 | AHR | 2.567652887 | 14.87146219 |
| NM_002247 | KCNMA1 | 2.566793296 | 14.87146219 |
| NM_002160 | TNC | 2.49357493 | 4.95297081 |
| NM_007190 | SEC23IP | 2.483504358 | 14.87146219 |
| NM_014751 | MTSS1 | 2.473598115 | 18.90170085 |
| AA906057 | AA906057 | 2.469146533 | 4.95297081 |
| NM_174907 | PPP4R2 | 2.447254704 | 17.40088182 |
| NM_001040441 | ZBTB8 | 2.443152652 | 19.34106852 |
| NM_018192 | LEPREL1 | 2.442702659 | 18.90170085 |
| THC2574606 | THC2574606 | 2.438142908 | 3.432321877 |
| NM_177968 | PPM1B | 2.430257094 | 3.432321877 |
| DB352127 | DB352127 | 2.424825247 | 14.36263262 |
| NM_145055 | C18orf25 | 2.415856913 | 12.11119291 |
| NM_000186 | CFH | 2.402621647 | 4.95297081 |
| NM_057159 | LPAR1 | 2.38878103 | 16.90104045 |
| NM_001017995 | SH3PXD2B | 2.361539024 | 3.432321877 |
| NM_014258 | SYCP2 | 2.353034052 | 16.39461008 |
| AK021874 | AK021874 | 2.334108923 | 16.39461008 |
| BC033331 | BC033331 | 2.321622362 | 16.39461008 |
| NM_006023 | CDC123 | 2.321074533 | 16.90104045 |
| BC033829 | BC033829 | 2.298202466 | 18.01968985 |
| NM_022482 | GZF1 | 2.298107974 | 16.90104045 |
| NM_033111 | N4BP2L2 | 2.295862765 | 18.01968985 |
| NM_052854 | CREB3L1 | 2.288554223 | 7.524705653 |
| NM_014413 | EIF2AK1 | 2.28612286 | 18.01968985 |
| NM_014624 | S100A6 | 2.277252504 | 14.36263262 |
| THC2668815 | THC2668815 | 2.257872563 | 16.90104045 |
| NM_016307 | PRRX2 | 2.240596479 | 12.22764669 |
| NM_000851 | GSTM5 | 2.169821964 | 4.95297081 |
| NM_001042576 | RRBP1 | 2.166407445 | 16.39461008 |
| NM_005746 | NAMPT | 2.163615408 | 18.01968985 |
| AI916036 | AI916036 | 2.153845851 | 14.87146219 |
| NM_001677 | ATP1B1 | 2.122556948 | 12.22764669 |
| NM_021213 | PCTP | 2.096517299 | 18.90170085 |
| NM_130386 | COLEC12 | 2.085327547 | 18.90170085 |
| NM_007033 | RER1 | 2.084230538 | 17.40088182 |
| AK074256 | SBNO1 | 2.041374058 | 17.40088182 |
| NM_001008397 | LOC493869 | 1.991325214 | 14.87146219 |
| NM_001008397 | LOC493869 | 1.989081645 | 16.39461008 |
| NM_001008397 | LOC493869 | 1.989004834 | 16.39461008 |
| NM_004048 | B2M | 1.985998314 | 18.90170085 |
| NM_001008397 | LOC493869 | 1.981914894 | 14.36263262 |
| NM_001008397 | LOC493869 | 1.970168725 | 16.39461008 |
| NM_001008397 | LOC493869 | 1.968429703 | 16.39461008 |
| U04815 | U04815 | 1.966734957 | 4.733282588 |
| NM_001008397 | LOC493869 | 1.956516181 | 16.39461008 |
| NM_001008397 | LOC493869 | 1.948094549 | 14.87146219 |
| NM_001008397 | LOC493869 | 1.945548517 | 16.90104045 |
| NM_001008397 | LOC493869 | 1.941638173 | 16.39461008 |
| AK001062 | AK001062 | 1.890723806 | 14.36263262 |
| NM_007361 | NID2 | 1.876797672 | 16.90104045 |
| CR613654 | CR613654 | 1.854739815 | 18.90170085 |
| BC111484 | BC111484 | 1.817765776 | 14.87146219 |
| NM_003316 | TTC3 | 1.805471704 | 14.87146219 |
| NM_007124 | UTRN | 1.797279972 | 16.90104045 |
| THC2568538 | THC2568538 | 1.790515801 | 19.34106852 |
| NM_000270 | NP | 1.783224669 | 18.01968985 |
| BQ365891 | BQ365891 | 1.772327749 | 5.754186676 |
| NM_000303 | PMM2 | 1.771228764 | 17.40088182 |
| NM_000430 | PAFAH1B1 | 1.708415106 | 18.90170085 |
| AY117690 | AY117690 | 1.70768676 | 18.90170085 |
| NM_015646 | RAP1B | 1.673522059 | 5.754186676 |
| NM_017801 | CMTM6 | 1.644959731 | 16.39461008 |
| NM_001037494 | DYNLL1 | 1.621288788 | 19.34106852 |
| AY374131 | MTHFD1L | 1.616992965 | 18.01968985 |
| NM_014372 | RNF11 | 1.565557166 | 12.98511153 |
| THC2533833 | THC2533833 | 1.515766769 | 16.39461008 |
| NM_003667 | LGR5 | 1.483683442 | 18.01968985 |
| NM_001044305 | SMAP1 | 1.474495287 | 18.90170085 |
| ENST00000361624 | COX1 | 1.470563641 | 18.01968985 |
| NM_001077489 | GNAS | 1.454729334 | 4.95297081 |
| NM_001077489 | GNAS | 1.428339832 | 14.36263262 |
| A_24_P186354 | A_24_P186354 | 1.427232111 | 16.39461008 |
| NM_015137 | EFR3A | 1.410868221 | 18.90170085 |
| NM_007178 | STRAP | 1.389667372 | 4.733282588 |
| NM_003901 | SGPL1 | 1.380326443 | 16.39461008 |
| BC014938 | BC014938 | 1.374174596 | 16.90104045 |
| NM_005766 | FARP1 | 1.356697859 | 18.01968985 |
| BX460543 | BX460543 | 1.351902483 | 17.40088182 |
| NM_005032 | PLS3 | 1.349121165 | 2.274911011 |
| NM_001675 | ATF4 | 1.330621251 | 16.39461008 |
| BX641010 | BX641010 | 1.304307762 | 19.34106852 |
| ENST00000379284 | ENST00000379284 | 1.258060971 | 18.01968985 |
| NM_004045 | ATOX1 | 1.234930377 | 14.36263262 |
| NM_007262 | PARK7 | 1.223931323 | 18.90170085 |
| NM_014262 | LEPREL2 | 1.200099294 | 0 |
| AL832190 | COG2 | 1.198639248 | 16.39461008 |
| AK098548 | AK098548 | 1.151977729 | 5.754186676 |
| ENST00000382819 | ENST00000382819 | 1.127577296 | 18.90170085 |
| **Negative genes (15 genes and 26 probes)** | | | |
| **Gene ID** | **Gene Name** | **Fold Change** | **q-value(%)** |
| AB023144 | SEZ6L | 0.154919497 | 2.794890671 |
| NM_006040 | HS3ST4 | 0.182769874 | 16.40479307 |
| NM_178824 | WDR49 | 0.197930268 | 16.90104045 |
| NM_057157 | CYP26A1 | 0.228085864 | 12.11119291 |
| AK075525 | FLJ14712 | 0.26872948 | 14.36263262 |
| BM678897 | BM678897 | 0.350986253 | 12.11119291 |
| NM_020789 | IGSF9 | 0.37090764 | 12.11119291 |
| CR608347 | HLA-B | 0.551247146 | 14.36263262 |
| CR608347 | HLA-B | 0.55464516 | 12.11119291 |
| CR608347 | HLA-B | 0.554954232 | 14.36263262 |
| CR608347 | HLA-B | 0.557935703 | 14.36263262 |
| CR608347 | HLA-B | 0.559999165 | 12.11119291 |
| CR608347 | HLA-B | 0.563637822 | 12.11119291 |
| CR608347 | HLA-B | 0.563836826 | 12.11119291 |
| CR608347 | HLA-B | 0.566294681 | 12.11119291 |
| CR608347 | HLA-B | 0.570649637 | 16.40479307 |
| CR608347 | HLA-B | 0.57117755 | 14.36263262 |
| NM_021109 | TMSB4X | 0.573429134 | 12.11119291 |
| NM_152304 | RAB42 | 0.576508782 | 16.40479307 |
| NM_152527 | SLC16A14 | 0.581325928 | 2.794890671 |
| AK091220 | HIST3H2BB | 0.656426194 | 17.40088182 |
| NR_001555 | GOLGA2LY1 | 0.685529659 | 12.22764669 |
| NM_002738 | PRKCB1 | 0.731040458 | 20.88517755 |
| NM_175575 | WFIKKN2 | 0.744169977 | 0 |
| NM_153606 | FAM71A | 0.768913736 | 14.87146219 |
| AT_nX_3 | AT_nX_3 | 0.809897092 | 16.90104045 |
| **Odontoblasts vs Secretory ameloblasts** | | | |
| **Negative genes (4 and 4 probes)** | | | |
| Gene ID | Gene Name | Fold Change | q-value(%) |
| NM_031889 | ENAM | 0.122755702 | 0 |
| NM_004319 | ASTN1 | 0.175137944 | 0 |
| NM_182681 | AMELX | 0.212559903 | 34.18467287 |
| AB023144 | SEZ6L | 0.382572847 | 34.18467287 |
| **Pre-secretory ameloblasts vs secretory ameloblasts** | | | |
| **Negative genes (4 genes and 5 probes)** | | | |
| Gene ID | Gene Name | Fold Change | q-value(%) |
| NM_004407 | DMP1 | 0.094046089 | 0 |
| NM_016519 | AMBN | 0.151480896 | 0 |
| NM_016429 | COPZ2 | 0.172171525 | 0 |
| NM_194318 | B3GALTL | 0.30065783 | 19.56785101 |
| A_24_P585198 | A_24_P585198 | 0.365472527 | 0 |
