# Supplementary material for: Towards Unraveling the Human Tooth Transcriptome: The Dentome
Source: PLoS One. 2015 Apr 7;10(4):e0124801. doi: 10.1371/journal.pone.0124801 (PMC4388651; doi:10.1371/journal.pone.0124801)
Supplement: S2 Table — (DOCX) [file pone.0124801.s003.docx]

**S2 Table. Top 20 differentially expressed canonical pathways between odontoblasts and pre-secretory ameloblasts.**

| **Ingenuity Canonical Pathways** | **-log(p-value)** | **Ratio** | **Molecules** |
| --- | --- | --- | --- |
| Intrinsic Prothrombin Activation Pathway | 3.84E00 | 1.14E-01 | COL1A2,COL1A1,COL11A2,COL3A1 |
| Hepatic Fibrosis / Hepatic Stellate Cell Activation | 3.44E00 | 4.79E-02 | COL1A2,COL1A1,FN1,PDGFRA,TGFB2,PDGFC,COL3A1 |
| Atherosclerosis Signaling | 3.12E00 | 4.41E-02 | COL1A2,COL1A1,CLU,COL11A2,PDGFC,COL3A1 |
| Phospholipase C Signaling | 2.89E00 | 3.08E-02 | RAP1B,GNAS,RALB,RHOU,RPS6KA3,ATF4,MEF2C,PRKCB |
| Dendritic Cell Maturation | 2.48E00 | 2.9E-02 | B2M,COL1A2,COL1A1,ATF4,COL11A2,COL3A1 |
| ERK5 Signaling | 2.46E00 | 6.15E-02 | SGK1,RPS6KA3,ATF4,MEF2C |
| Corticotropin Releasing Hormone Signaling | 2.44E00 | 3.68E-02 | RAP1B,GNAS,ATF4,MEF2C,PRKCB |
| Sphingosine-1-phosphate Signaling | 2.42E00 | 4.17E-02 | GNAS,PDGFRA,RHOU,SMPD3,PDGFC |
| Guanosine Nucleotides Degradation III | 2.21E00 | 9.09E-02 | NT5E,PNP |
| Urate Biosynthesis/Inosine 5'-phosphate Degradation | 2.14E00 | 9.09E-02 | NT5E,PNP |
| Adenosine Nucleotides Degradation II | 2.14E00 | 7.69E-02 | NT5E,PNP |
| ILK Signaling | 2.07E00 | 3.12E-02 | FN1,TMSB10/TMSB4X,RHOU,VIM,ATF4,PDGFC |
| Melanocyte Development and Pigmentation Signaling | 2.01E00 | 4.4E-02 | GNAS,RPS6KA3,ATF4,KIT |
| Xanthine and Xanthosine Salvage | 2E00 | 1.11E-01 | PNP |
| Adenine and Adenosine Salvage I | 2E00 | 1.11E-01 | PNP |
| Purine Nucleotides Degradation II (Aerobic) | 1.96E00 | 5.71E-02 | NT5E,PNP |
| Glioma Signaling | 1.83E00 | 3.57E-02 | IGF2,PDGFRA,PDGFC,PRKCB |
| Guanine and Guanosine Salvage I | 1.7E00 | 1.11E-01 | PNP |
| Sulfate Activation for Sulfonation | 1.7E00 | 1.25E-01 | PAPSS2 |
| NGF Signaling | 1.7E00 | 3.39E-02 | RAP1B,RPS6KA3,ATF4,SMPD3 |
