# Supplementary material for: Towards Unraveling the Human Tooth Transcriptome: The Dentome
Source: PLoS One. 2015 Apr 7;10(4):e0124801. doi: 10.1371/journal.pone.0124801 (PMC4388651; doi:10.1371/journal.pone.0124801)
Supplement: S3 Table — (DOCX) [file pone.0124801.s004.docx]

**S3 Table. Top 20 different upstream regulators between odontoblasts and pre-secretory ameloblasts.**

| **Upstream Regulator** | **Predicted Activation State** | **Activation z-score** | **p-value of overlap** | **Target molecules in dataset** | **Mechanistic Network** |
| --- | --- | --- | --- | --- | --- |
| WNT3A |  | 1.605 | 3.84E-11 | AHR,ATP1B1,COL11A2,COL1A1,DCN,FN1,LGR5,MEF2C,NES,PDGFRA,RHOU,SOX9,SP7,TGFB2,TNC | SMAD1,TP53,WNT3A,heparin |
| Alpha catenin | Inhibited | -3.405 | 1.05E-10 | COL1A1,COL1A2,COL3A1,IGF2,LAMA4,LUM,PDGFRA,SGK1,TNC,VIM,WIPF1,ZEB2 |  |
| TGFB1 | Activated | 3.549 | 4.98E-09 | ACVRL1,AHR,ATF4,B2M,CDKN1C,CLU,COL1A1,COL1A2,COL3A1,DCN,DYNLL1,EGR3,ENPP1,FN1,GNAS,IGF2,KIT,LIFR,MEF2C,NAMPT,NT5E,PLS3,PNP,PPFIBP2,RALB,RASL11B,RGCC,SGK1,SOX9,SPOCK1,STRAP,TGFB2,TGFBI,TNC,VIM,ZEB2 | AHR,AR,CEBPB,EGR1,HIF1A,IL6,NFKBIA,NFkB (complex),RELA,SP1,SP3,TGFB1,TNF,TP53,VHL,beta-estradiol |
| GLI1 | Activated | 3.274 | 1.05E-08 | CLU,COL1A1,EGR3,IGF2,MEF2C,NES,PAPSS2,PCDH18,PDGFRA,SALL1,SP7,TNC |  |
| FGF2 |  | 0.952 | 3.67E-08 | ATF4,COL1A1,COL1A2,COL3A1,DCN,ENPP1,FN1,IGF2,NES,PDGFC,PDGFRA,SOX9,TGFB2,TMSB10/TMSB4X,VIM | CBL,CEBPB,CTNNB1,EGR1,FGF2,HRAS,JUN,KLF4,NFKBIA,PRL,RELA,RHOA,SMAD1,SMAD3,SMAD4,SP1,SP3,TNF,TP53 |
| PRL | Activated | 2.670 | 4.69E-08 | B2M,CLU,COL1A1,COL1A2,COL3A1,DCN,FN1,IGF2,PDGFRA,RALB,TMSB10/TMSB4X,VIM | AHR,AR,CBL,CEBPB,CTNNB1,D-glucose,EGR1,IGF1,JUN,NFE2L2,NFKBIA,PRL,SP1,TP53,beta-estradiol |
| NFKBIA |  | 1.324 | 5.08E-08 | CDKN1C,CLU,COL1A2,COL3A1,DCN,FN1,GSTM5,IGF2,LIFR,PARK7,SGK1,SOX9,SPOCK1,SYCP2,TGFB2,TMSB10/TMSB4X,TNC | AR,CEBPB,CTNNB1,EGR1,FGF2,IL6,JUN,NFKBIA,NFkB (complex),RELA,SP1,SP3,TNF,TP53 |
| Tretinoin |  | 0.763 | 6.76E-08 | AHR,AKAP12,ATF4,COL1A1,COL1A2,COL3A1,CYP26A1,DCN,DYNLL1,FN1,IGF2,KIT,LAMA4,MEF2C,NES,NID2,PDGFRA,PLS3,PNP,PRKCB,RALB,SMPD3,SOX9,SP7,TGFB2,TGFBI,TMSB10/TMSB4X,TNC,VIM,WIPF1,ZEB2 | AR,CEBPB,CTNNB1,EGFR,EGR1,FGF2,HIF1A,NFKBIA,RELA,SMAD3,SMAD4,SMAD7,SP1,SP3,TGFB1,TNF,TP53,tretinoin |
| streptozocin | Activated | 2.316 | 2.12E-07 | ATP1B1,CLU,COL1A1,COL3A1,FN1,KIT,NT5E,PDGFRA,PPARGC1A,RPS6KA3,VIM | CBL,CEBPB,CTNNB1,EGR1,GATA4,IGF1,MEF2C,NFKBIA,RELA,SMAD3,SMAD4,SP1,SP3,TNF,TP53,streptozocin |
| IGF2BP1 | Activated | 2.219 | 2.45E-07 | COL1A1,COL1A2,IGF2,LUM,TNC |  |
| MSTN |  | 0.200 | 2.67E-07 | FN1,IGF2,MEF2C,PPARGC1A,TNC,VIM | MEF2C,MSTN,MYOD1 |
| SHH | Activated | 3.104 | 2.80E-07 | FN1,FOXF1,IGF2,MEF2C,NES,PDGFRA,SALL1,SOX9,SP7,TGFB2 | BMP2,JUNB,KLF4,MEF2C,MYOD1,RUNX2,SHH,SMAD1,SMAD6 |
| SMAD7 | Inhibited | -2.374 | 3.09E-07 | COL1A1,COL1A2,COL3A1,DCN,FN1,NID2,SOX9,TGFB2,TGFBI | EGR1,EP300,GATA4,KLF4,MAP2K3,NFE2L2,NFkB (complex),RHOA,RUNX2,SMAD1,SMAD3,SMAD4,SMAD6,SMAD7,SP1,TGFBR1 |
| MEF2C |  | -0.342 | 8.56E-07 | COL1A1,COL1A2,COL3A1,MEF2C,PPARGC1A,SP7,VIM | GATA4,MEF2C |
| dimethylnitrosamine | Activated | 2.449 | 1.37E-06 | COL1A1,COL1A2,COL3A1,IGF2,RALB,VIM | CEBPB,CTNNB1,EGR1,HIF1A,NFKBIA,RELA,SMAD7,SP1,SP3,TGFB1,TNF,dimethylnitrosamine |
| PTCH1 |  | -1.432 | 1.41E-06 | FOXF1,IGF2,MTSS1,PDGFRA,SOX9 | MYOD1,PTCH1,SHH |
| CR1L | Inhibited | -2.236 | 1.79E-06 | COL1A1,COL1A2,COL3A1,LUM,TNC |  |
| TP53 | Activated | 2.246 | 1.96E-06 | AKAP12,ATF4,CLU,COL1A1,COL1A2,COL3A1,EGR3,FN1,GSTM5,IGF2,KCNMA1,NAMPT,NID2,PARK7,PDGFRA,PPM1B,PPP4R2,PRKCB,PRRX2,SEL1L,SGK1,SGPL1,SP7,TGFB2,TGFBI,TMSB10/TMSB4X,VIM | AR,CEBPB,EGR1,EP300,FGF2,JUN,NFKBIA,PPARG,SMAD3,SMAD4,SP1,TGFB2,TP53 |
| HRAS |  | -0.122 | 1.96E-06 | B2M,CLU,COL1A1,COL1A2,COL3A1,ENPP1,FN1,IGF2,MTHFD1L,NID2,PDGFC,PDGFRA,PLS3,PRKCB,TGFB2,TMSB10/TMSB4X,VIM | AR,CEBPB,CTNNB1,EGR1,EP300,HRAS,JUN,NFKBIA,SMAD3,SMAD4,SMAD7,SP1,TP53 |
| beta-estradiol |  | 1.519 | 2.06E-06 | AHR,ATP1B1,BLOC1S6,CDC123,CDKN1C,CDR1,CLU,COL1A1,COL1A2,COL3A1,COLEC12,DCN,DYNLL1,EGR3,FN1,IGF2,KIT,LUM,NAMPT,PAPSS2,PDGFRA,PMM2,PNP,PRKCB,RER1,SGK1,SOX9,SPOCK1,TGFB2,VIM,ZEB2 | AR,CBL,CEBPB,CTNNB1,EGR1,HIF1A,IL6,JUN,NFKBIA,NFkB (complex),PRL,SMAD3,SMAD4,SMAD7,SP1,TGFB1,TP53,beta-estradiol |
